# Feminist Political Economy of Health: Current Perspectives and Future Directions

**DOI:** 10.3390/healthcare9020233

**Published:** 2021-02-22

**Authors:** Iffath Unissa Syed

**Affiliations:** Institute of Health Policy, Management and Evaluation (IHPME), Institute for Pandemics, Dalla Lana School of Public Health, University of Toronto, Toronto, ON M5T 3M6, Canada; iffath.syed@utoronto.ca

**Keywords:** health policy, social policy, sociology, feminist health studies, feminist epistemology

## Abstract

Feminist political economy of health is a term that has emerged as a result of research that has combined and connected a feminist political economy lens with a focus on health disparities of women. This paper provides an overview of the literature from the work of feminist medical sociologists and feminist health scholars that have shaped the concept of feminist political economy of health. The analysis indicates that while women have experienced health inequities inside the healthcare system, there are also significant health disparities that are experienced outside the healthcare system due to women’s social, economic, political, and cultural conditions. Given that there are dual crises with respect to the COVID-19 pandemic as well as social movements pushing for change, further work that uses intersectional approaches is advocated.

## 1. Introduction

Feminist political economy is a popular term that has been used in various disciplines and various fields of studies, such as sociology, humanities, political science, environmental studies, work/labor studies, and public health. More recently, the term has been updated and utilized by medical sociologists and public health practitioners, and it has been referred to as feminist political economy of health. Feminist political economy of health suggests that material and cultural discrimination against girls and women are the primary factors that influence their social conditions and health [1,2]. 

Feminist political economy of health has its roots in feminism and the work of feminist scholars. Feminism is concerned with gender inequalities that arise from a system of patriarchy [3]. Feminist scholars and feminists argue that society is gendered in such a way that women and men have fundamentally different experiences and access to power and privilege [3]. Feminists have both criticized and expanded upon materialist approaches in representing and considering women’s perspectives [3]. Feminist sociologists argue that the economic, social, and political issues for women arise as a result of social and political histories that were developed and written exclusively by men, and from the standpoint of men rather than women [3,4,5,6]. Feminist materialist scholarship extends the ideas of materialism by connecting market relations with domestic ones [7]. Feminist political economy frameworks also focus on equity for women [8]. Scholars examine the economic needs of the family, the work of women in the home and in labor markets, and relations within workplaces. Feminist materialist scholarship also examines tensions related to women’s paid and unpaid work, such as how production and reproduction affects women’s lives [9]. For instance, women’s reproduction and unpaid caregiving roles can modulate the extent to which women participate in the paid economy, which can then affect their material, social, and political conditions, their overall life circumstances, and their health. Context becomes important, and there is a focus on the interactions between the micro (individual), meso (structural/organizational), and macro (global/international) levels [10]. 

Feminist perspectives have been influential in the fields of public health, as well as in medical sociology, and in the sociology of women’s health and illness [3]. Feminist political economy approaches that are applied to health research have helped to explain the processes that make women vulnerable to health inequities at a variety of levels. The first has to do with the health care needs of women. For instance, women have different health needs than men and require diversity in health services [1,2]. Yet, biomedical research and biomedicine are often applied to women in unfair ways that have health-compromising consequences for women [11]. For example, women may experience pregnancy, birth, and different types of health issues during their aging and life course compared to men. However, health research and health care services often do not fit with women’s needs [9]. Furthermore, the Physician Health Study of coronary heart disease also excluded women from their trials because coronary heart disease was thought of as a male problem, despite the fact that half a million women died of it each year in the USA [1], and despite that women’s risk of it and other chronic illness is influenced by a number of factors [12]. Studies also show that in some societies, girls and women experience inequities in the healthcare system due to discrimination because boys and men are valued more than girls and women [13,14]. 

## 2. Women Can Experience Health Inequities Both Inside and Outside the Healthcare System

Although many women experience health inequities from within the healthcare system, as mentioned in the example above, they also experience health inequities that are separate from (or outside the scope of) the health care system. For example, marriage or having children can prevent women from pursuing continuing education, and also impact their paid work obligations [2], which can modulate rates of poverty, women’s material conditions, and accordingly, their health equity. For instance, research from the United States has shown that while recent cohorts of women have entered the labor market more than their predecessors, the number of weekly hours is low due to “an influx of part-time workers” ([15], p. 109). Other studies show that the female labor force continues to be concentrated in traditional sectors of their employment, such as education, healthcare, social services, and culture [16], which tend to pay them less than men, i.e., women experience horizontal segregation [17]. Within specific occupational groups, women also tend to work less frequently than men in positions with higher occupational hierarchy (i.e., managerial jobs), alluding to vertical segregation, as well (ibid). 

Working women can also be susceptible to illness and absence from work due to family obligations. This phenomenon, consisting of a dual paid/unpaid work–family interface is often dubbed a “double burden” [18,19]. For example, women tend to provide the majority of care in both public and private environments [20]. They also maintain the health and lives of those in their households, families, and communities through unwaged and low-waged invisible work (ibid). This is especially true in the health care sector, which comprises of over 80% women as workers [18]. The double burden of paid work in the labor force combined with unpaid work in the home can make women more vulnerable to poorer health than men, and it can also cause stress and fatigue, which have spiraling, health-compromising effects on their wellbeing [21,22]. Unfortunately, the double burden of women’s work often remains invisible because women are expected to be responsible for social reproduction, yet paradoxically, this social reproduction overlaps both the public and private realms [18]. 

### 2.1. Feminist Materialism

Feminist materialists who are focused on an analysis of health argue that women’s health and wellbeing are directly affected by determinants of health, such as income and social status, because women are often paid lower wages than men [1,2,23]. This economic disparity can influence women’s access to healthy food and nutrition, their participation in health-modifying and health-impacting behaviors, as well as access to health/medical, social, dental and other types of care. Another problem that affects women’s health and wellbeing is that women are often sex segregated in the labor market. This means that women who participate in labor markets may do so as a reserve supply/army of labor on either a daily, weekly, seasonally, or part-time basis to respond to demand and overproduction in manufacturing, retail, service, and other sectors [24,25,26,27]. 

Feminist materialists also argue that the health problems women experience are related to their discrimination and disadvantage while they carry out the gendered activities that make up their daily lives [1,2,28]. Specifically, the dual demands of women’s work in the home and the labor market have a direct effect on the way women participate in the workforce as well as on the sex segregation of women’s work and women’s wages [2]. For example, if a job involves care work, it is most often performed by a woman, it is classified as unskilled, and therefore, it is lower-paid than that of men in caregiving occupations [29]. These types of disparities in women’s working conditions result in gender inequities in income and wealth, which make women vulnerable to poverty and also vulnerable to health problems. 

There are, however, political situations and contexts in which women are able to balance these dual demands, participate equitably in the labor market, and benefit from public spending [30]. This context began with the first wave of feminism, and women’s right to vote, which started with the Western states in the United States, and eventually enabled the politics of redistribution, since women vote for high taxation and high social protection through spending more than men [30]. Research indicates that in social democratic regimes such as Sweden, Norway, Denmark, and Finland, redistributive policies have encouraged women to gain meaningful employment; provided family-oriented services, such as childcare and home care to help women integrate into the paid labor market; provided generous non-means-tested social services and transfers, and unemployment compensation for single mothers; and helped support women’s health and participation in government [31]. On the opposite spectrum, societies that have a “[t]olerance for corruption” and exhibit Nietzschean behavior have limited or no social mobility, ethics, rule of law, and the ruling elites increase spending at the expense of public spending on education, schools, teachers, medical facilities, and health [32] (p. 276). 

Although many feminists have embraced materialism, acknowledging that the accumulation of profits and wealth among the Bourgeoisie class (now capitalist class) has resulted from the labor power advanced by women and men from the Proletariat class, i.e., the working class, the assumption that “Wage labor rests exclusively on the competition between laborers,” [26] (p. 21) becomes problematic. The reason for this is because it raises Malthusian and Ricardian principles of scarcity of food and land, respectively, due to mass population growth and urbanization, which requires a restoration of equilibrium. Marx predicted a communist revolution, but it was only carried out in Russia and China, while other countries adopted social democratic principles [33]. Furthermore, certain phenomena were unanticipated by materialist perspectives such the abolishment of slavery; the replacement of slavery with racialized labor diasporas [34]; technological innovation such as automobiles; and globalization, which shifted power and capital dramatically including to those who now own oil wells and gas commodities [33].

Research indicates that there are also ethical, moral and material implications of women’s work, which also compromise their health and wellbeing, and this is based on particular stereotypes. For instance, in women’s work in the health sector, and specifically within long term care (LTC), there is a perception that good women care for their families and others, either uncompensated or low-paid, and in doing so, they attain feminine moral worth [20]. As a result, while such types of women’s work in the home or in the labor market are morally and ethically elevated in this regard, they can be detrimental to women’s material conditions because they are often invisible [35], unpaid or underpaid, unregulated, un-supervised, undervalued, and they can be characterized by long hours, and can have dull, repetitive, and isolating working conditions that can perpetuate health inequities [2]. The literature shows that care work, for example, might also be undeclared and undocumented [36]. Furthermore, care workers frequently feel they are unseen, unheard, and accordingly, they are unhappy [37]. Material conditions such as employment opportunities, wages, and scarcity of day-care also constrain women’s roles and can confine them to work primarily in the home, even if ideas about women’s place in the home changes [2]. 

Feminist health scholars challenge the assumption that maleness and femaleness starts and usually ends with sex differences in reproductive systems [38,39,40]. The biological differences between women and men go beyond the obvious ones related to reproductive systems, and also include genetic, hormonal, metabolic and other variations [1,41]. Furthermore, while there are obvious differences between male and female patterns of sickness and health, with some health problems stemming from biological differences, these differences are more complex than pure biology [1,2]. Many feminists, therefore, reject these arguments about biological determinism and biological models, and they suggest that the differences between men and women, and among different groups of women, are beyond biology and include things such as socio-economic status, class, culture, discrimination, and racism. 

One of the debates in feminist materialist scholarship is that certain forms of material conditions seem to be advantageous for women, but they might actually be disadvantageous. For example, part-time work has been advocated as a solution to the problem of balancing paid work and care responsibilities by allowing women (especially mothers) to balance their time in caregiving while also participating in paid work [42]. However, the material reality is that in part-time work, women may have low wages, they often have few opportunities for training and career progression, they have inequitable access to employment benefits, and they often do not receive paid leave [2]. The reasons for these inequities experienced by part-time women-workers are due to various sex-stereotypes about women such as having high absenteeism, being uncommitted to their work, being unproductive, inability to be career oriented, or wanting few responsibilities and pressures [2]. Part-time work and part-time wages might also make women submissive and more dependent on men, coined “housewifization”, because women’s wages and power within the family might become lower than that of men [1,2]. Thus, advocating part-time work or other one-size-fits-all approaches for women are not necessarily ideal practices. 

Feminist political economy work is helpful in explaining the material differences in health by gender, and it goes beyond gender in multiple ways; but, it was initially limited in its anti-racism analysis and critique, the latter of which only emerged in the second and third waves of feminism [8]. The particular concerns of women (and men) of color that were initially neglected under materialism began to change during the latter feminist social movements. It is now widely accepted that there are alternative and unique perspectives that can be offered when gender is analyzed in ways that intersect with race, class, ability or disability, age, and other social identities that lead to further marginalization, and that men and women’s lives are embedded in this reality [43,44]. For example, disabled people’s experiences are not only limited to material abuses such as physical or emotional violence and poverty, but go beyond these experiences to include forms of social exclusion, oppression, objectification, and alienation [45]. Such experiences also include neo-racism and democratic racism [46]. Indeed, many racialized women experience significant occupational segregation and earn lower wages than other groups [47]. Research in the United States demonstrates that the actual disparity between black women’s wages and white women’s wages is 17% [48].

Another example of conflict is the competition between jobs taken up by low-waged temporary or seasonal migrant and racialized workers versus unemployed Canadian citizens who possess the same skills. These groups are pitted against each other while the capitalist class makes gains by exploiting either side. This was exactly the scenario that occurred in British Columbia, Canada in which the International Union of Operating Engineers and the Construction and Specialized Workers’ Union asked the federal court Justice, Douglas Campbell to grant an injunction to stop Chinese foreign workers from arriving in Canada [49]. The unions were concerned that foreign workers were being recruited to work for HD Mining Ltd., while Canadian miners were not given a fair opportunity to work at the mine first [50]. Yet, foreign workers also need to work in order to survive and thrive, and often have to sustain their families abroad. Many of the above-noted perspectives have been inspired by critical feminist scholars and critical race theorists, who advocate intersectional approaches to research, which is described in the next section.

### 2.2. Intersectionality and Theories of Knowledge

Intersectionality as a conceptual framework can extend the work of feminist approaches that are otherwise limited in their analysis of racism and other forms of discrimination. In other words, intersectionality frameworks may be promising alternatives to a one-size-fits-all approach [51,52]. For instance, they may help to frame racialized women’s unique or different experiences and lived-realities, as well as other markers of difference, such as urban versus rural contexts. In addition to intersectional frameworks, feminist health scholars have contributed to feminist epistemology, ontology, and methodology. 

Epistemology refers to the study of knowledge [51]. Epistemology is associated with how the inquirer or researcher, who wishes to understand a particular subject, creates knowledge about these matters through research and other inquiries [53]. Examples of epistemology include the epistemology of positivism and feminist epistemology. The epistemology of positivism has its disciplinary roots in the natural sciences [54]. Positivists believe in empiricism, which is the idea that observation and measurement is the core of the scientific endeavor [55]. Positivism assumes and strives for objectivity, in which the researcher attempts to minimize or exclude political values, subjective impressions, and partial accounts that might bias their findings [54]. Feminist epistemology is a way of thinking that suggests that gender should influence the practice of acquiring knowledge and also the analysis of that knowledge [56]. Feminist epistemology identifies the ways in which the dominant conception, acquisition, and justification of knowledge systematically disadvantage women and other subordinated groups [56].

Ontology refers to theories of being [51] (p. 10). The ontology of critical theories, such as gender/feminist political economy and ethnicity/anti-racism), is historical realism [57]. In historical realism, reality is shaped by social, political, economic, cultural, ethnic, and gender values (ibid). 

Methodology is the theory and analysis that informs research [51]. Methodology often refers to the study of/discourses about systematic, theoretical analyses of the methods applied to a given field of study. In other words, methodology is focused on the specific ways, or the methods that are used to try to understand the world [55,57] (As indicated by scholars [53] (p. 126), “methodology is about the kinds of research tools that can be employed to acquire worthwhile knowledge about the world”. However, methodology is distinct from methods, the latter of which refers to the techniques or tools used to gather data [51]. For instance, positivist methodology incorporates the use of the scientific method and emphasizes experimentation in an attempt to discern natural laws through direct manipulation and observation [55]. Positivist methodologies identify scientific findings through observable and often quantifiable evidence [54]. It is believed that the world and the universe are operated by laws of cause and effect that can be applied to the unique approach of the scientific method [55]. In other words, positivist methodologies often emphasize the use of surveys and other quantitative approaches for knowledge acquisition and generation.

Unlike the quantitative methodological emphasis in the school of positivism, research that uses critical theories often has a methodological emphasis on a dialogic/dialectical approach [57] Researchers who use critical theories would likely employ qualitative methods, which are inductive and without predetermined hypotheses [58]. They would likely ask different questions than positivists, and those questions would be addressed in alternative ways [59,60,61]. For example, the what, how, or why of phenomena are asked rather than how many or how much [54] and this provides insights into the dimensions of experience, and helps to add to the completeness of answers to the questions that are asked [61,62]. 

Scholars using a feminist political economy approach have grounded their studies in critiques of gender and class ideologies, with a methodological approach that conforms to the tradition of historical realism [53,63]. In other words, qualitative methods are often employed in gathering data, although quantitative methods have also been used to draw on statistics that have established the female-dominated care workforce, women’s unequal position in the health-care field, and to reveal systemic discrimination against women [9,18,29]. Scholars recognize the importance of context and experiences, and seek a wide variety of sources for information and evidence [9]. This context is local and familial, as well as global and capitalist [9]. Accordingly, researchers’ roles, propositions, actions, and end-goals might aim to emancipate, empower, and socially transform people’s situations towards more equity and justice [57].

While feminist methodological research practices have endorsed qualitative methods, particular ones are encouraged more than others. For example, the work of some feminists supports interview methods as opposed to only using observational methods [5]. The strength of interview methods is that it prevents objectifying the research subject as an “Other” [5], and gives them voice. It is proposed that researchers can also improve this experience beyond interview practice, and explore relations that are embedded in our everyday world [5]. 

Feminist materialist scholarship has also contributed a wealth of knowledge to the epistemological and methodological approaches in gathering social, economic, and health data. For instance, “[m]aterialists contend that both bodies and ideas must be understood within the context of material conditions” [2] (p. 171). Consequently, biology affects and is affected by social, economic, and political conditions. In other words, if one were to inquire as to whether or not biological factors/gene expression or social/economic/political conditions contribute to a person’s health and wellbeing, then materialists would contend that biology/gene expression is influenced by social, economic, and political conditions. 

Feminist perspectives offer a number of epistemological and methodological critiques of mainstream health research, demonstrating the bias in how women are diagnosed, treated, and even studied. For example, the study and evidence used in the dominant research paradigms privilege quantitative methods, data, and numbers, such as randomized control trials (RCTs), number of nurses per population, and number of Caesarean procedures per doctor [9]. However, they can lead to certain biases. Indeed, RCTs have often been based on trials conducted on 70-kilogram adult males rather than females [9]. Furthermore, a study of coronary heart disease (CHD) entitled the Physician Health Study excluded women from their trials because CHD was thought of as a male problem, despite the fact that women are also at risk of CHD and other chronic illness [12], and despite that half a million women die of CHD each year in the USA [1]. 

Feminist epistemological perspectives also critique how knowledge that is gained may define how woman are perceived in society and how these perceptions can affect women’s paid and unpaid roles. For instance, culture, societal norms, and attitudes generate ideologies that define feminine and masculine work [64,65,66]. Ideas, ideologies, cultural norms, and attitudes about women get socially accepted over long periods of time and manifest themselves as health inequities [1,2]. As a result of the socialization process, internalization of biased ideas, and the social exclusion of women based on sexual stereotypes, women may become vulnerable to social problems. This vulnerability may worsen their health in a number of ways. For example, what is perceived as women’s work includes comforting, cooking, feeding, bathing, toileting, record-keeping, cleaning, laundering, management and supervision, as well as other tasks [18]. Many of these tasks have been carried out traditionally in the home and other private spheres, making them invisible. The problem arises when meals have to be prepared on time, and when children are looked after on-demand, which has the effect of restricting women’s abilities to work outside the home, making them vulnerable to income inequalities and health inequities [2].

Cultural factors are extremely important for women’s health and wellbeing, along with societal norms, values, and attitudes towards girls and women, which shape their material conditions and health outcomes. For example, research suggests that women are more risk averse than men, and these differences are actually present early in life [67] possibly due to cultural conditions. In certain cultures, the notion of family honor can become deadly when honor-killings are carried out against girls and women who are unfairly accused of breaking from tradition. In addition, there are also harmful cultural practices such as female genital cutting that deprive women of sexual pleasure while also simultaneously exposing them to chronic gynecological conditions. Lastly, in some conservative Middle Eastern cultures, women are not allowed to be without the presence of male guardians, which limits employment opportunities, thereby reducing women’s opportunity to work outside of the home [68]. 

A new problem that has now emerged is how to care for women and other vulnerable people during crises such as the COVID-19 pandemic. Addressing this problem requires thinking about all of the ways in which their health is affected. Another area of opportunity would be to examine the experience of groups who are racialized minorities, such as Muslim women. Recent research has examined how the human rights and health of racialized women are shaped by state policies and structural/organizational decisions, which impact racialized women who often have little say in the decisions that affect their lives [69,70]. It would be interesting to examine socioeconomic disparities among these marginalized groups using intersectional approaches and feminist epistemology and methodology, which were mentioned in this paper.

## 3. Conclusions and Prospects

This paper has discussed the ways in which feminist scholarship has shaped and defined the concept of feminist political economy of health. While these terms have been around for quite some time, emerging scholarship can always add new ways to think about feminist political economy of health. New and emerging research should be undertaken that examines how women’s work, women’s material/economic, social, and cultural conditions, and health disparities, are affected by contemporary crises. This will be particularly important given the current research emerging from the conditions during the COVID-19 pandemic as well as the ‘Black Lives Matter’ social movement in liberal democratic societies.

## Data Availability

No new data were created or analyzed in this study. Data sharing is not applicable to this article.
